# Region-Specific Effects of Immunotherapy With Antibodies Targeting α-synuclein in a Transgenic Model of Synucleinopathy

**DOI:** 10.3389/fnins.2018.00452

**Published:** 2018-07-04

**Authors:** Martin Kallab, Marcos Herrera-Vaquero, Malin Johannesson, Fredrik Eriksson, Jessica Sigvardson, Werner Poewe, Gregor K. Wenning, Eva Nordström, Nadia Stefanova

**Affiliations:** ^1^Division of Neurobiology, Department of Neurology, Innsbruck Medical University, Innsbruck, Austria; ^2^BioArctic AB, Stockholm, Sweden

**Keywords:** immunotherapy, α-synuclein, autophagy, animal model, α-synuclein clearance

## Abstract

Synucleinopathies represent a group of neurodegenerative disorders which are characterized by intracellular accumulation of aggregated α-synuclein. α-synuclein misfolding and oligomer formation is considered a major pathogenic trigger in these disorders. Therefore, targeting α-synuclein species represents an important candidate therapeutic approach. Our aim was to analyze the biological effects of passive immunization targeting α-synuclein and to identify the possible underlying mechanisms in a transgenic mouse model of oligodendroglial α-synucleinopathy. We used PLP-α-synuclein mice overexpressing human α-synuclein in oligodendrocytes. The animals received either antibodies that recognize α-synuclein or vehicle. Passive immunization mitigated α-synuclein pathology and resulted in reduction of total α-synuclein in the hippocampus, reduction of intracellular accumulation of aggregated α-synuclein, particularly significant in the spinal cord. Lowering of the extracellular oligomeric α-synuclein was associated with reduction of the density of activated iba1-positive microglia profiles. However, a shift toward phagocytic microglia was seen after passive immunization of PLP-α-synuclein mice. Lowering of intracellular α-synuclein was mediated by autophagy degradation triggered after passive immunization in PLP-α-synuclein mice. In summary, the study provides evidence for the biological efficacy of immunotherapy in a transgenic mouse model of oligodendroglial synucleinopathy. The different availability of the therapeutic antibodies and the variable load of α-synuclein pathology in selected brain regions resulted in differential effects of the immunotherapy that allowed us to propose a model of the underlying mechanisms of antibody-aided α-synuclein clearance.

## Introduction

α-synuclein is an attractive target for disease modification in synucleinopathies including Parkinson’s disease (PD), dementia with Lewy bodies (DLB), and multiple system atrophy (MSA) (Brundin et al., 2017) Experimental data from transgenic models based on the overexpression of α-synuclein support the causative role of α-synuclein pathology as a trigger of neurodegeneration (Stefanova and Wenning, 2015; Stefanova, 2017; Ko and Bezard, 2017; Koprich et al., 2017). Recent propagation studies proposed prion-like properties of α-synuclein which may contribute to the progression of neurodegeneration (Steiner et al., 2018). For all these reasons α-synuclein pathology emerges as a valid therapeutic target in synucleinopathies.

Immunotherapy is being pursued as one of several strategies to reduce α-synuclein pathology in PD and MSA, where early clinical trials are in progress (Brundin et al., 2017; Koga and Dickson, 2017). Previous work has demonstrated that active immunization against α-synuclein in PD and MSA transgenic mice mitigates motor deficits, reduces α-synuclein pathology, modulates neuroimmune responses and leads to neuroprotection (Mandler et al., 2014, 2015; Villadiego et al., 2018). There is now increasing evidence from PD models that passive immunization with antibodies against pathogenic α-synuclein species may promote the clearance of α-synuclein and reduce neurodegeneration (Masliah et al., 2011; Games et al., 2014; Lindstrom et al., 2014; El-Agnaf et al., 2017; Spencer et al., 2017). It is suggested that antibodies against α-synuclein may act by promoting clearance of α-synuclein via the autophagy-lysosomal pathway (Masliah et al., 2011) or via microglia-dependent degradation (Bae et al., 2012). Importantly, α-synuclein antibodies may interact with both intracellular and extracellular α-synuclein, and therefore interfere with intracellular aggregate formation, cell-to-cell spread and induction of pro-inflammatory responses (Lopes da Fonseca et al., 2015; Lee and Lee, 2016).

In the current study we aimed to analyze the effects of passive immunization with an antibody targeting α-synuclein in the PLP-α-syn transgenic mouse, which was engineered to express human full-length α-synuclein under the proteolipid protein (PLP) promoter in oligodendrocytes (Kahle et al., 2002). This transgenic mouse is considered a model of MSA that features intra-oligodendroglial α-synuclein inclusion formation, α-synuclein-triggered microglial activation, and neurodegeneration.(Stefanova et al., 2005, 2007; Stefanova and Wenning, 2015)

## Materials and Methods

### Animals and Treatment

A recombinant α-synuclein antibody, rec47, which preferentially binds oligomeric species (Lindstrom et al., 2014), was produced using a CHOK1SV GSKO Glutamine Synthetase (GS) system as previously described (Lindstrom et al., 2014). The antibody was purified using a custom made HiScale 26/10 Protein G-sepharose column (GE Healthcare) and SEC-purified over a HiLoad 26/60 Superdex 200 prep grade column (GE Healthcare, 17-1071-01). The final buffer was PBS (Dulbecco’s PBS, Gibco) and the concentration was determined by measuring A_280_ nm on a Nanodrop instrument with IgG settings. The preference of rec47 for α-synuclein oligomers was confirmed by inhibition ELISA (Lindstrom et al., 2014).

Homozygous male and female transgenic PLP-α-synuclein mice (MGI:3604008) overexpressing human α-synuclein under the PLP promoter (Kahle et al., 2002) and age- and sex-matched non-transgenic C57Bl/6 background mice were used in this study. All animals were bred and housed in a temperature-controlled room under a 12/12 h dark/light cycle, with free access to food and water and under special pathogen free conditions in the animal facility of the Medical University of Innsbruck. All experiments were performed in accordance with the Austrian law under permission BMWFW-66.011/0125-WF/V/3b/2015.

At the age of 4 months, 10 mice per group started receiving bi-weekly intraperitoneal injections of anti-α-synuclein antibody rec47 (20mg/kg b. w.) or a corresponding volume of vehicle (PBS) for a period of 12 weeks. The choice of the treatment dosing was selected based on the experience in previous studies (Lindstrom et al., 2014). The age of initiation of treatment was chosen to cover a time window in which PLP-α-synuclein mice show progressive GCI pathology, microglial activation and neuronal loss in SNc without strong motor phenotype presented yet (Refolo et al., 2018).

### Tissue Sampling

Mice were transcardially perfused with 20 ml PBS for 5 min under deep thiopental anesthesia 1 week after the last injection. The brains and spinal cords were quickly removed. The left hemisphere was dissected in sub-regions (forebrain, hippocampus, midbrain, cerebellum, lower brainstem). Each sub-region as well as the most rostral cervical spinal cord were immediately frozen on dry ice, stored at -80°C and further used for biochemical analysis. The right hemisphere and the caudal cervical spinal cord were immersion fixed in 4% paraformaldehyde at 4°C overnight, cryoprotected with 30% sucrose and slowly frozen and stored at -80°C and further used for histological analysis.

### Homogenization and Tissue Extraction

The tissue extraction was sequential, starting with homogenization in TBS (20 mmol/l Tris and 137 mmol/l NaCl, pH 7.6) followed by extraction in TBS or TBS/1% Triton X-100 (TBS/T). The TBS/T pellets were thereafter dissolved in either 1% SDS or 70% formic acid (FA). In more details, the snap-frozen tissue was homogenized in TBS supplemented with protease and phosphatase inhibitors (Roche, Mannheim, Germany) using PreCellys at 1:3-1:10 volume ratios, depending on the weight of tissue. The homogenate was divided and mixed with an equal volume of TBS or TBS/T, followed by centrifugation at 16,000 *g* for 1h (+4°C). The supernatants, corresponding to soluble and membrane associated α-synuclein (TX-soluble fraction) were collected and stored at -80°C until analyses. The TBS/T pellets were thereafter extracted in 1% SDS or 70% FA. The SDS extracted samples were spun down at 16,000 *g* for 1 h at ambient temperature, the supernatant was collected (TX-insoluble/SDS soluble fraction) and stored at -80°C until analyses of phosphorylated α-synuclein. The TBS/T pellets extracted in 70% FA were spun at 100,000 *g* for 1 h (+4°C), the supernatant was collected and stored at -80°C until analyses of insoluble α-synuclein (TX-insoluble/FA soluble fraction). For the following analysis, the levels of the analytes were compensated to a final concentration of 1:10 or expressed as g analyte/g tissue. All further analyses were performed in a blinded fashion.

### α-synuclein Measurements

The anti-α-synuclein antibody, synuclein-1 (BD) for detection of total α-synuclein was coated on a 96-well standard MSD plate (0.5 μg/ml) in PBS. Free binding sites were blocked by incubation with 1% Blocker A solution (MSD). Samples and standard (recombinant α-synuclein, BioArctic AB or α-synuclein-HNE complexes) were allowed to interact with the coated antibody. α-synuclein species, bound to the capture antibody, were detected by adding the oligoclonal rabbit anti-α-synuclein antibody FL140 (0.2 μg/ml, Santa Cruz Biotechnology) followed by MSD SULFO-TAG anti-rabbit IgG (MSD) or biotin-conjugated mAb38F (0.5 μg/ml) followed by Streptavidin labeled MSD SULFO-TAG (0.5 μg/ml) and 2x MSD Read Buffer T addition according to manufactures description (MSD). SECTOR Imager 600 (MSD) was used to detect the emitted light that correlates to the amount of α-synuclein in the samples. The plates were washed with PBS-T (0.05%) between each incubation step.

### Antibody Availability in the CNS

Rec47 was measured in TBS/T homogenates prepared as described. 96-well standard MSD plates were coated with 0.5 μg/ml recombinant α-synuclein (BioArctic AB) in PBS. Before addition of sample and standard, (rec47), free binding sites were blocked by incubation with 1% Blocker A (MSD). Bound antibodies were detected by a goat anti-mouse IgG antibody (Southern Biotech) followed by streptavidin labeled MSD SULFO-TAG and 2x MSD Read Buffer T according to manufactures description (MSD). SECTOR Imager 600 (MSD) was used to detect the emitted light that correlates to the amount of antibody in the sample. The plates were washed with PBS-T (0.05%) between each incubation step.

### Western Blotting for LC3b

Brain sub-regions and spinal cords were separately homogenized in TBS buffer (w/v, 1:10) with a complete protease inhibitor cocktail (Roche, Mannheim, Germany) using a tissue grinder and further protein quantification, separation, and immunoblotting were performed according to standard protocols. Shortly, equal amounts of protein were loaded on 15% SDS-PAGE gels and separated by gel electrophoresis. Proteins were then transferred to PVDF membranes (Merck Millipore, Billerica, MA, United States). After incubation in 2% Amersham ECL Blocking Agent (GE Healthcare Life Sciences, Boston, MA, United States) in PBS supplemented with 0.2% Tween20 for 1 h, the membranes were incubated with primary antibodies [LC3 (1:1000, Cell Signaling, Danvers, MA, United States); β-III-tubulin (1:500, Abcam, Cambridge, United Kingdom)] overnight at 4°C on an orbital shaker. Membranes were incubated for 1 h at room temperature with the horseradish-peroxidase (HRP)-conjugated secondary antibody (1:20000, GE Healthcare Life Sciences). Bands were visualized by enhanced chemiluminescent reagent (Biozym, Hessisch Oldendorf, Germany). Immunoblots were scanned (Fusion FX, Vilber Lourmat, Marne-la-Vallée, France) and densitometry was measured with the FusionCapt Advance software (Vilber Lourmat, Marne-la-Vallée, France). LC3b-II band intensities were normalized to the loading control β-III-tubulin, and normalized values were further statistically analyzed.

### Histopathology

Fixed hemispheres and spinal cords were cut on a cryotome (Leica, Nussloch, Germany) at 40 μm thickness of the sections. Immunohistochemistry on free-floating sections was performed using standard protocols and the following antibodies: anti-aggregated α-synuclein (5G4, Linaris, Germany), anti-phosphorylated α-synuclein (pS129, ab51253, Abcam, United Kingdom), anti-Iba1 (ab108539, Abcam, United Kingdom), anti-tyrosine hydroxylase (TH, AB152, Millipore, Germany), anti-CD68 (MCA1957GA, Serotec, United Kingdom), biotinylated anti-mouse, anti-rat and anti-rabbit IgG (respectively, BA-1000, BA-9400 and BA-2001, Vector Laboratories, United States). The reaction was enhanced with ABC Elite kit (PK-6100, Vector Laboratories, United States) and visualized with 3, 3′-diaminobenzidine. Cresyl violet was used for counterstaining of sections immunostained with anti-Iba1 antibody. For co-localization analysis, immunofluorescence for pS129 and LC3b (Cell Signaling, Leiden, Netherlands) followed by Alexa 488- and Alexa 594-conjugated secondary antibodies (Thermo Fisher Scientific, Rockford, IL, United States) was performed.

### Image Analysis

All analyses were performed by an examiner blinded to the treatment and genotype of the animals. The StereoInvestigator Software (MicroBrightField) provided to a Nikon Eclipse 80i microscope with motorized stage and high resolution digital camera was applied. A low power objective lens (2x, SPlan) was used to delineate the borders of the areas of interest. Actual object counting was done using a 100x objective (NA 0.75) using the automated meander scan procedure. The data were presented as object density per mm^2^ and thereafter used for the analysis. Dopaminergic neuronal numbers in SNc were determined using the optical fractionator as previously described (Refolo et al., 2018).

To study microglial activation, Iba1- and CD68-immunostaining optical density was assessed in microphotographs for the regions of interest shot at constant camera and light settings at the same microscope. The data used for the analysis were presented as mean relative optical density with correction for background signal as previously described (Stefanova et al., 2012). Next we defined the number of Iba1-positive microglial cells with activated morphological profile [previously described as B, C, and D type (Sanchez-Guajardo et al., 2010; Refolo et al., 2018)] per area and represented the data as cells per mm^2^. In detail, type B cells were characterized by their hyperramified processes and larger cell body, type C cells presented with enlarged cell body and shortening and thickening of the processes, and type D microglia showed amoeboid form.

### Confocal Microscopy

Three-dimensional stacks were acquired with an SP8 confocal microscope (Leica Microsystems, Wetzlar, Germany) using a HC PL APO CS2 63x, 1.3 NA glycerol immersion objective. Imaging was performed using WLL with excitation lines for Alexa 488 at 498 nm and for Alexa 594 at 590 nm. Fluorescence emission was detected in sequence 1 from 503 to 576 nm (Alexa 488) and in sequence 2 from 594 to 742 nm (Alexa 594). Images were acquired using the Leica LAS X 3.1.1 acquisition software (Leica Microsystems). Image deconvolution was performed using Huygens Professional software (Scientific Volume Imaging, Hilversum, Netherlands). The Colocalization Analyzer was used to define the degree of overlap of signals in 3D.

### Statistical Analysis

All data are presented as mean ± SEM. To test statistical significance of the treatment, the data sets were analyzed with Student’s t-test or Mann–Whitney test depending on the distribution of the values, and if not indicated otherwise. Two-way ANOVA was used when two variables (genotype/treatment or sub-region/treatment) were considered. Statistical significance was set at *p* < 0.05 considering two-tailed confidence interval. Correlations were assessed by linear regression analysis. The statistical analysis was performed with the GraphPad Prism Software.

## Results

### Passive Immunization of PLP-α-synuclein Mice Resulted in Region-Specific Amelioration of α-synuclein Pathology

**FIGURE 1:**
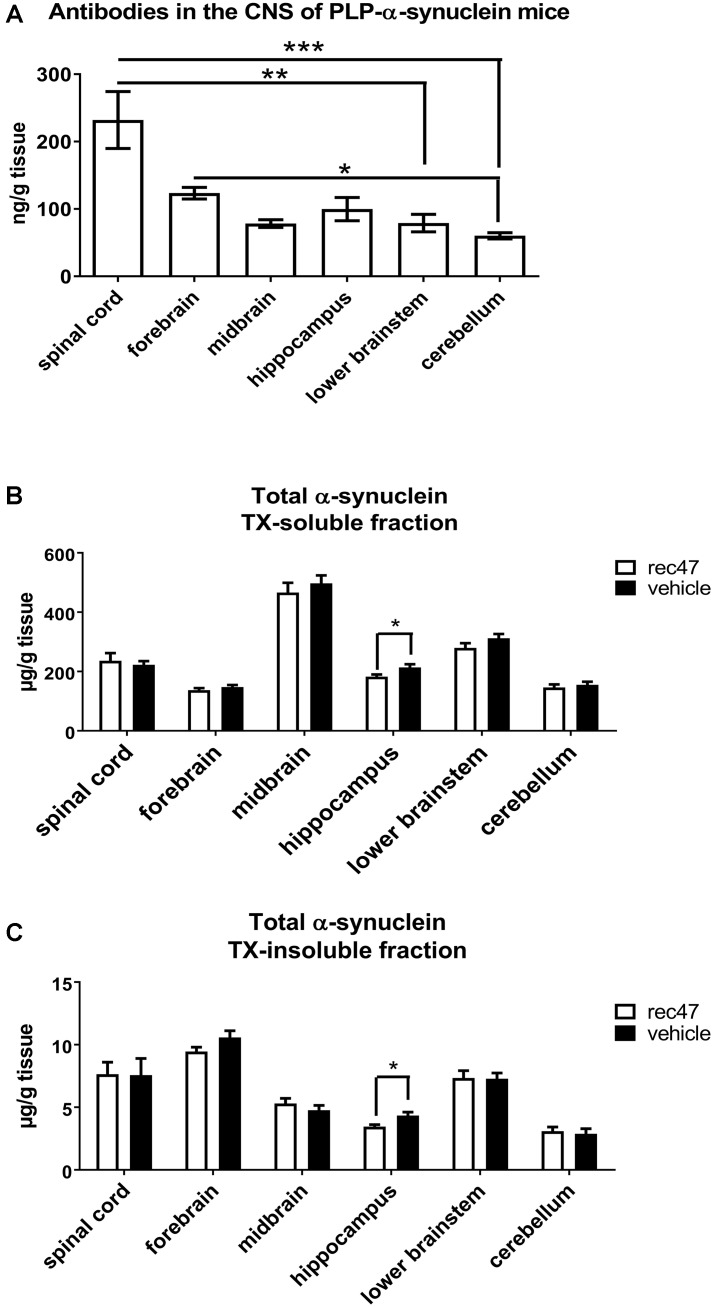
**(A)** Availability of antibodies in CNS regions after immunotherapy of PLP-α-synuclein mice (*n* = 7 for all groups). **(B)** Level of soluble and **(C)** insoluble total α-synuclein species in the CNS of PLP-α-synuclein mice (*n*_vehicle_ = 8, *n*_rec47_ = 9) tested by ELISA (^∗^*p* < 0.05; ^∗∗^*p* < 0.01; ^∗∗∗^*p* < 0.001).

The passive immunization in PLP-α-synuclein mice over a period of 3 months resulted in no significant differences in the survival of the animals. We assayed in parallel all CNS samples from PBS and rec47 treated mice for levels of antibodies by ELISA. In vehicle-treated animals or non-transgenic controls receiving rec47, no signal was detected, suggesting a specific IgG response to the rec47 therapy only in PLP-α-synuclein mice. In PLP-α-synuclein mice receiving passive immunization, the rec47 exposure in the CNS showed region-specific differences of IgG levels. The level of antibodies was significantly higher in the spinal cord as compared to lower brainstem (*p* < 0.01) and cerebellum (*p* < 0.001). The cerebellum showed also significantly lower levels of antibodies as compared to the forebrain (*p* < 0.05). The availability of antibodies in the CNS of PLP-α-synuclein mice was distributed as follows: spinal cord > forebrain > hippocampus > midbrain > lower brainstem > cerebellum, **Figure 1A**).

The levels of total α-synuclein were measured in TX-soluble (**Figure 1B**) and TX-insoluble/FA soluble (**Figure 1C**) fractions of the forebrain, midbrain, hippocampus, cerebellum, brain stem and spinal cord of PLP-α-synuclein receiving anti-α-synuclein antibodies or vehicle. A significant decrease of total α-synuclein was detected in the TX-soluble and TX-insoluble fractions of the hippocampus in the rec47 treated group (**Figures 1B,C**). Oligomeric α-synuclein levels were variably present in the different sub-regions of the CNS of PLP-α-synuclein mice receiving vehicle (spinal cord < hippocampus < lower brainstem < midbrain < cerebellum < forebrain, **Table 1**). Overall lower levels of soluble α-synuclein oligomers were detected in PLP-α-synuclein mice receiving rec47 as compared to vehicle, however, sub-region post-hoc analysis failed to reach statistical significance (**Table 1**).

**Table 1:** Levels of oligomeric α-synuclein (ng per g tissue) in different sub-regions of the CNS of PLP-α-synuclein mice undergoing treatment with either vehicle or rec47 antibody.

Treatment group	Spinal cord	Forebrain	Midbrain	Hippocampus	Brainstem	Cerebellum
Vehicle (*n* = 8)	21.1 ± 3.6	49.5 ± 15.6	41.3 ± 9.5	37.5 ± 11.4	40.1 ± 10.2	45.1 ± 16.9
rec47 (*n* = 9)	12.6 ± 0.8	38.7 ± 4.0	40.4 ± 3.2	31.6 ± 2.7	29.6 ± 3.6	26.7 ± 1.8

*No significant effect of the treatment was idendtified in the studied CNS sub-regions (n > 0.05 for all), despite a trend of overall reduction of the levels of oligomeric α-synuclein after therapy with rec47 of PLP-α-synuclein mice.*

**FIGURE 2:**
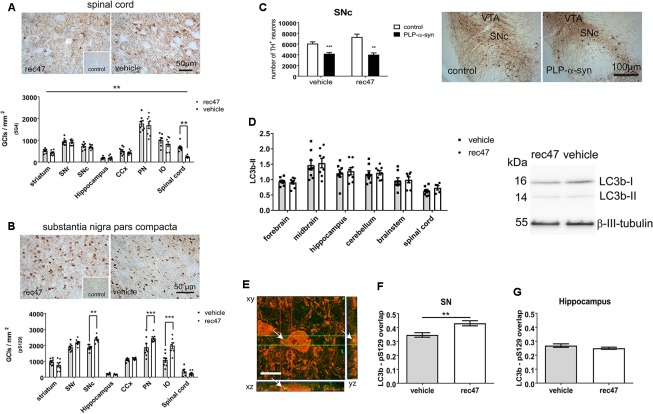
**(A)** Immunohistochemistry for oligomeric α-synuclein (5G4) in PLP-α-synuclein mice, the inset shows the lack of staining in control non-transgenic mice. **(B)** Immunohistochemistry for phosphorylated α-synuclein (pS129) in PLP-α-synuclein mice, the inset shows the staining pattern in control non-transgenic mice. **(C)** Dopaminergic neurons positive for TH in SNc estimated by stereology (SNc, substantia nigra pars compacta; VTA ventral tegmental area). **(D)** Western blotting for LC3b; LC3b-II signal is normalized to β-III-tubulin level. **(E)** Confocal microscopy to explore the level of colocalization of LC3 (Alexa 488, green, arrows) and pS129 (Alexa 594, red), scale bar, 5 μm. **(F)** LC3 signal in pS129-positive cells in the substantia nigra (SN) of PLP-α-synuclein mice receiving rec47 immunotherapy or vehicle. **(G)** LC3 in pS129-positive cells in the hippocampus of PLP-α-synuclein mice (^∗∗^*p* < 0.01; ^∗∗∗^*p* < 0.001).

To address selectively the effects of immunotherapy on the intracellular accumulation of α-synuclein in the brains of PLP-α-synuclein mice, we applied immunohistochemistry. To detect aggregated α-synuclein in glial cytoplasmic inclusions (GCI)-like structures which are typically found in the PLP-α-synuclein brain we used the 5G4 antibody as previously described (Kovacs et al., 2012; Brudek et al., 2016). The density of GCI-like profiles was significantly affected by the treatment as assessed by two-way ANOVA [effect of treatment: *F*_1,102_ = 6.798, *p* < 0.01; effect of region: *F*_7,102_ = 76.48, *p* < 0.001; interaction *F*_7,102_ = 0.9264, *p* > 0.05]. The treatment-induced change of the density of 5G4-positive GCIs proved significant after the *post hoc* Bonferroni correction in the spinal cord (**Figure 2A**).

The phosphorylation of the intracellular α-synuclein was measured by the density of cells with pS129-positive intracellular accumulation. We found both effects of treatment (*F*_1,102_ = 17.06, *p* < 0.001) and region (*F*_7,102_ = 103.1, *p* < 0.001) as well as interaction between the two variables (*F*_7,102_ = 7.74, *p* < 0.001) by two-way ANOVA. Interestingly, *post hoc* Bonferroni test demonstrated a significant increase of intracellular α-synuclein phosphorylation in SNc, pontine nuclei (PN), and inferior olives (IO) but not in the other tested regions (**Figure 2B** and Supplementary Figure 1).

Since SNc neurons show a specific vulnerability to the α-synuclein pathology in the PLP-α-synuclein mouse model (Stefanova et al., 2005, 2007; Fernagut et al., 2014; Refolo et al., 2018), we addressed whether the changes induced by rec47 immunotherapy (increased intracellular α-synuclein phosphorylation, **Figure 2B**) may affect nigral neuronal loss. No change in the neuronal loss in SNc of PLP-α-synuclein mice versus control non-transgenic mice was seen after therapy with rec47 antibodies (effect of genotype: *F*_1,19_ = 43.04, *p* < 0.0001; effect of treatment: *F*_1,19_ = 1.799, *p* = 0.1957; interaction: *F*_1,19_ = 3.141, *p* = 0.0924, **Figure 2C**).

### Mechanisms of Intracellular α-synuclein Clearance After Immunotherapy in PLP-α-synuclein Mice

Previous experimental evidence suggests that macroautophagy is a major mechanism of protein degradation after immunotherapy targeting α-synuclein (Masliah et al., 2011). Furthermore, phosphorylation of α-synuclein can be related to preparation of the protein for degradation and clearance (Tenreiro et al., 2014). We sought to determine whether changes in the autophagy pathway can be detected in PLP-α-synuclein mice receiving anti α-synuclein antibodies. Western blot analysis showed no significant change in the LC3b-II levels in any of the brain sub-regions studied (**Figure 2D**). Next, we used confocal microscopy to identify the density of LC3-positive particles in pS129-immunopositive cells in selected brain regions (**Figure 2E**). The data show a significant increase in LC3 signal in pS129-positive cells in SNc in immunized versus control mice (**Figure 2F**), while this is not the case in the hippocampus – a region where no change in the level of α-synuclein phosphorylation was detected (**Figure 2G**).

### Changes of the Microglia Activation Profile After Passive Immunization of PLP-α-synuclein Mice

**FIGURE 3:**
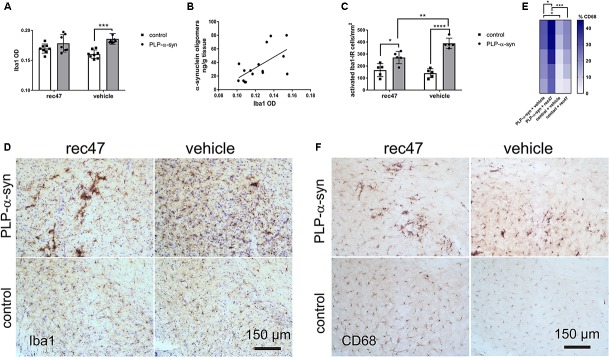
**(A)** Microglial activation measured by Iba1-immunoreactivity optical density in PLP-α-synuclein and control mice (^∗^*p* < 0.05, ^∗∗∗^*p* < 0.001). **(B)** Correlation between the level of α-synuclein oligomers and microglial activation in the hippocampus of PLP-α-synuclein mice (*R*^2^ = 0.381, *p* < 0.01), the analysis includes both immunized and non-immunized mice. **(C)** Microglial activation measured by the number of Iba1-immunoreactive cells with activated morphology per mm^2^ in PLP-α-synuclein mice and controls. **(D)** Representative microphotographs of Iba1 immunohistochemistry in SNc of control and PLP-α-synuclein mice receiving rec47 or vehicle (counterstaining with cresyl violet). **(E)** Heat map representation of the percentage CD68-positive microglia out of the total Iba1-positive microglia in the four experimental groups (^∗^*p* < 0.05, ^∗∗∗^*p* < 0.001). **(F)** Representative microphotographs of CD68 immunohistochemistry in SNc of control and PLP-α-synuclein mice receiving rec47 or vehicle.

Previous studies propose that microglia can be activated by oligomeric α-synuclein released into the extracellular space (Fellner et al., 2011, 2013; Fellner and Stefanova, 2013; Sanchez-Guajardo et al., 2015). Confirming previous observations, (Stefanova et al., 2007) we found a significantly higher Iba1 immunosignal in PLP-α-synuclein mice as compared to wild type controls receiving vehicle (**Figure 3A**). The difference was lost after anti-α-synuclein antibody treatment. Significant correlation between the level of α-synuclein oligomers and microglial activation was detected in PLP-α-synuclein mice (**Figure 3B**); however, no such correlation was found between microglial activation and phosphorylation of intracellular α-synuclein (*R*^2^ = 0.07, *p* = 0.34).

Next, the number of Iba1-immunoreactive microglia with activated morphology type B, C, or D (Sanchez-Guajardo et al., 2010; Refolo et al., 2018) in PLP-α-synuclein mice as compared to wild type controls was determined. Treatment with rec47 in PLP-α-synuclein mice resulted in significant reduction in the number of activated (type B, C, and D) microglial cells (**Figures 3C,D**). Interestingly, after immunotherapy in PLP-α-synuclein there was a clear increase of the clusters of type C and D cells, while B type was predominant in the vehicle treated animals (**Figure 2D**). Since C and D type morphology of microglia may represent increased phagocytic activity, we tested the expression of CD68, a microglia lysosomal marker. The percentage of CD68-immunosignal significantly increased in PLP-α-synuclein mice receiving rec47 and was linked to the clusters of microglia observed in the Iba1 immunostaining (**Figures 3E,F**).

## Discussion

Our study provides evidence for biological effects of passive immunization with antibodies targeting α-synuclein in PLP-α-synuclein mice. Immunotherapy with rec47 antibody resulted in an overall trend of reduction of α-synuclein levels with decrease of GCI-density in the CNS. In the current experiment *post hoc* sub-regional analysis confirmed significant changes in α-synuclein pathology after immunotherapy in the hippocampus and the spinal cord. Finally, a shift of α-synuclein-induced microglial activation in PLP-α-synuclein mice toward a phagocytic phenotype was observed (Stefanova et al., 2007).

Oligomeric α-synuclein species are considered to precede the formation of amyloid fibrils of the intracellular aggregates in PD, DLB and MSA and to account for α-synuclein toxicity (Bourdenx et al., 2017; Wong and Krainc, 2017). For this reason, targeting oligomeric α-synuclein forms is considered a major candidate therapeutic approach in these disorders. In the PLP-α-synuclein mouse model, oligomeric high molecular weight α-synuclein species have been previously demonstrated (Kahle et al., 2002; Bassil et al., 2016, 2017; Venezia et al., 2017). Hence, the model provides a valid tool to test pre-clinically the biological effects of passive vaccines targeting preferentially α-synuclein oligomers as rec47 used in the current study (Lindstrom et al., 2014). The effects that we identified in the PLP-α-synuclein mouse after 3 months of rec47 immunization were region-specific. We hypothesize that the sub-regional efficacy may depend on the levels of penetrating antibodies and the levels of pathologic forms of α-synuclein in each sub-region.

**FIGURE 4:**
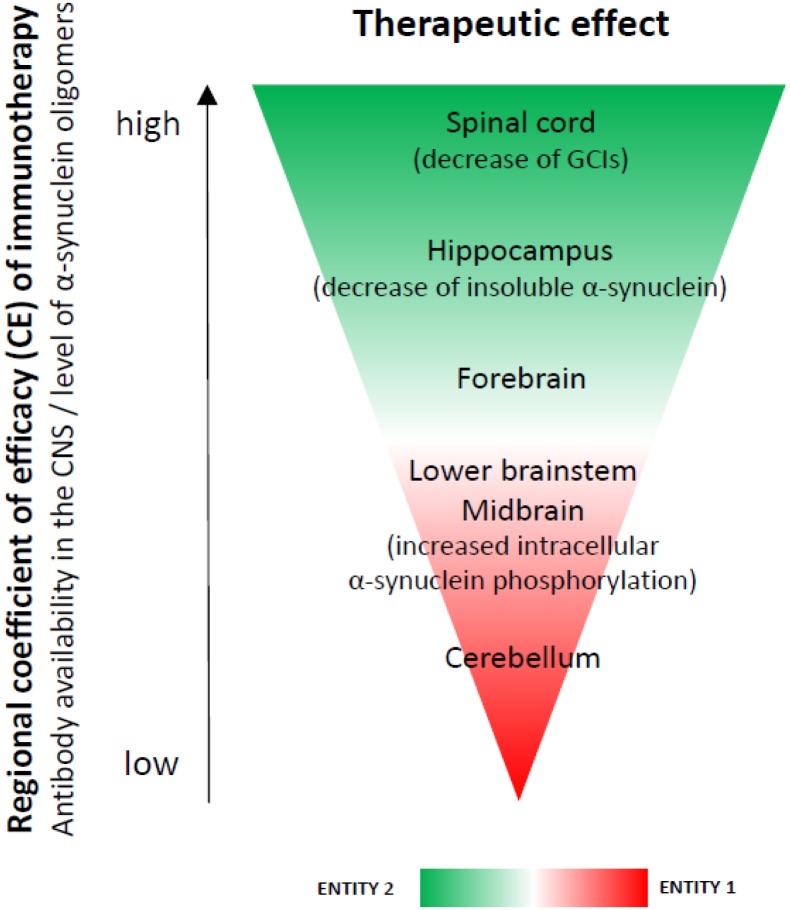
A hypothesis for a regional coefficient of efficacy (CE) after immunotherapy. The regional CE was calculated as a ratio between the antibody availability in the CNS and the level of oligomeric α-synuclein. An expected CE was distributed as follows: spinal cord > hippocampus > forebrain > lower brainstem > midbrain > cerebellum. We observed increased phosphorylation of intracellular α-synuclein in the brain stem (SNc, pontine nuclei, inferior olives), i.e., a region with lower CE (entity 1), but not in the spinal cord, forebrain or hippocampus– regions with higher CE (entity 2) after immunotherapy. We propose that these two entities may represent different stages of the dynamics of α-synuclein clearance after rec47 immunotherapy. While in entity 2 it is possible to already measure GCI reduction and reduction of insoluble α-synuclein, entity 1 may be useful to document preceding events before the actual removal of the toxic α-synuclein species.

We found region-specific CNS availability of the antibodies used for immunotherapy, confirming previous reports (Lindstrom et al., 2014). A possible explanation for this phenomenon may be the heterogeneity of the blood-brain and blood-spinal cord barrier (Wilhelm et al., 2016). The higher availability of antibodies in the spinal cord may be due to the higher permeability of the blood-spinal cord barrier as shown previously (Wilhelm et al., 2016). The blood-spinal cord barrier has been demonstrated to have fewer pericytes and reduced tight junction protein expression which increase permeability compared to the blood-brain barrier (Bartanusz et al., 2011; Winkler et al., 2012). On the other hand, the levels of α-synuclein were lowest in the spinal cord followed by the hippocampus. If a hypothetical efficacy coefficient of the immunotherapy per region is calculated as the ratio between the mean availability of antibodies versus the mean level of α-synuclein oligomers per region, the result will give expected efficacy score of spinal cord (11) > hippocampus (2.7) > forebrain (2.5) > lower brainstem (2.0) > midbrain (1.9) > cerebellum (1.3) (**Figure 4**). In concert with this hypothesis, the spinal cord was the CNS region where GCI density was significantly reduced in PLP-α-synuclein mice receiving immunotherapy, and the hippocampus showed a significant lowering of insoluble α-synuclein.

**FIGURE 5:**
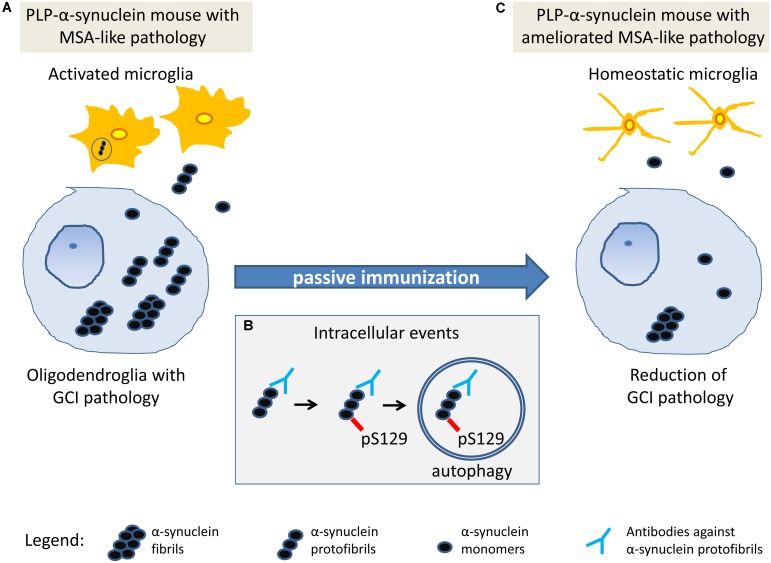
Working hypothesis on the mechanism of action of passive immunization with rec47 in PLP-α-synuclein mice. **(A)** At baseline in PLP-α-synuclein mice, oligodendroglia present with intracellular accumulation of α-synuclein oligomers that seed the aggregation of insoluble α-synuclein fibrils. Soluble oligomers released in the extracellular space trigger microglial activation that partly contributes to the clearance of α-synuclein. **(B)** Rec47 antibodies bind to α-synuclein oligomers and trigger increased phosphorylation of the protein which is further degraded by autophagy. **(C)** The immunotherapy with rec47 of PLP-α-synuclein mice results in clearance of α-synuclein oligomers leading to lowering of the intracellular seeding of α-synuclein aggregates (glial cytoplasmic inclusions, GCIs) and reduction of α-synuclein-induced microglial activation.

The mechanism of antibody-mediated clearance of intracellular α-synuclein aggregates is not completely understood. Earlier studies suggest that autophagy-lysosomal pathways may be involved (Masliah et al., 2011). We observed increased phosphorylation of intracellular α-synuclein in the brain stem (SNc, pontine nuclei, inferior olives), i.e., a region with lower antibody availability (entity 1), but not in the spinal cord, forebrain or hippocampus– regions with higher antibody concentration (entity 2) after immunotherapy. We propose that these two entities may represent different stages of the dynamics of α-synuclein clearance after rec47 immunotherapy. While in entity 2 it is possible to already measure GCI reduction, entity 1 may be useful to document preceding events before the actual removal of the toxic α-synuclein species. No significant overall changes in autophagy were measurable by immunoblotting, similar to a previous report in a PD model (El-Agnaf et al., 2017). However, we detected increase of intracellular α-synuclein phosphorylation linked to activation of autophagy (LC3 signal) in single cells of entity 1 (SNc), but not in entity 2 (hippocampus). Therefore, the detected selective increase of intracellular phosphorylation of α-synuclein is suggested to be an event related to the protein clearance (Tenreiro et al., 2014) at cellular level and may represent an earlier step in the pathways triggered by the antibody leading toward clearance of α-synuclein after immunotherapy (**Figure 5**).

Several studies indicate that microglia get activated in the presence of oligomeric α-synuclein and may participate in its clearance (Zhang et al., 2005; Austin et al., 2006; Stefanova et al., 2011; Fellner et al., 2013). In the PLP-α-synuclein mouse model, the presence of α-synuclein in oligodendrocytes leads to early activation of microglia (Stefanova et al., 2007). Therefore, microglial activation may reflect the levels of pathogenic α-synuclein species in the extracellular space in the PLP-α-synuclein mouse brain. Furthermore, microglia may show different activation profiles which may correspond to different functional phenotypes (Sanchez-Guajardo et al., 2010; Refolo et al., 2018), e. g. related to toxic pro-inflammatory responses or linked to beneficial phagocytic activity associated with the clearance of α-synuclein (Stefanova et al., 2011). Passive immunization with rec47 led to reduction of the density of Iba-1-positive microglia with activated morphology, pointing toward reduction of the levels of extracellular α-synuclein. Similar reduction of microglial activation after passive immunization targeting oligomeric forms of α-synuclein was shown in a PD models (El-Agnaf et al., 2017; Rockenstein et al., 2018), further corroborating the effects of immunotherapy in α-synucleinopathies and supporting the suggested here mechanisms (**Figure 5**). Interestingly, in PLP-α-synuclein mice treated with rec47 we found in addition a shift in the activated morphology of microglia from hyperramified type B toward profiles with shorter processes and larger amoeboid cell bodies (C and D type), i. e. a morphological phenotype associated with increased phagocytic activity. This notion was confirmed by the detection of increase percentage of CD68-positive microglia in PLP-α-synuclein mice treated with rec47. Altogether, the current results support the role of specifically activated microglia by the immunotherapy toward phagocytic phenotypes contributing for the clearance of pathogenic α-synuclein species from the extracellular space.

In summary, the data presented here further support the potential of immunotherapy targeting α-synuclein for the treatment of synucleinopathies. The effects observed in the current study of immunization of PLP-α-synuclein mice within a period of 3 months are mild and region-specific. However, the study provides insights into the biological mechanisms and the efficacy of the approach, supporting previous observations (Masliah et al., 2011; Bae et al., 2012; Lindstrom et al., 2014; Spencer et al., 2017).

## Author Contributions

MK, MH-V, MJ, and JS contributed to the execution of the experiments, wrote the first draft of the manuscript and its review. FE, WP, and GKW contributed to the discussion and interpretation of the data and review of the manuscript. EN and NS contributed to the conception of the study, the statistical analysis and interpretation of the data, and wrote and review of the manuscript.

## Conflict of Interest Statement

MJ, FE, JS, and EN are employees of BioArctic AB. The remaining authors declare that the research was conducted in the absence of any commercial or financial relationships that could be construed as a potential conflict of interest.

## References

[B1] Austin S. A. Floden A. M. Murphy E. J. Combs C. K. (2006). Alpha-synuclein expression modulates microglial activation phenotype. *J. Neurosci.* 26 10558–10563. 10.1523/JNEUROSCI.1799-06.2006 17035541 PMC6674709

[B2] Bae E. J. Lee H. J. Rockenstein E. Ho D. H. Park E. B. Yang N. Y. (2012). Antibody-aided clearance of extracellular alpha-synuclein prevents cell-to-cell aggregate transmission. *J. Neurosci.* 32 13454–13469. 10.1523/JNEUROSCI.1292-12.2012 23015436 PMC3752153

[B3] Bartanusz V. Jezova D. Alajajian B. Digicaylioglu M. (2011). The blood-spinal cord barrier: morphology and clinical implications. *Ann. Neurol.* 70 194–206. 10.1002/ana.22421 21674586

[B4] Bassil F. Canron M. H. Vital A. Bezard E. Li Y. Greig N. H. (2017). Insulin resistance and exendin-4 treatment for multiple system atrophy. *Brain* 140 1420–1436. 10.1093/brain/awx044 28334990 PMC6248513

[B5] Bassil F. Fernagut P. O. Bezard E. Pruvost A. Leste-Lasserre T. Hoang Q. Q. (2016). Reducing C-terminal truncation mitigates synucleinopathy and neurodegeneration in a transgenic model of multiple system atrophy. *Proc. Natl. Acad. Sci. U.S.A.* 113 9593–9598. 10.1073/pnas.1609291113 27482103 PMC5003293

[B6] Bourdenx M. Koulakiotis N. S. Sanoudou D. Bezard E. Dehay B. Tsarbopoulos A. (2017). Protein aggregation and neurodegeneration in prototypical neurodegenerative diseases: examples of amyloidopathies, tauopathies and synucleinopathies. *Prog. Neurobiol.* 155 171–193. 10.1016/j.pneurobio.2015.07.003 26209472

[B7] Brudek T. Winge K. Rasmussen N. B. Bahl J. M. Tanassi J. Agander T. K. (2016). Altered alpha-synuclein, parkin, and synphilin isoform levels in multiple system atrophy brains. *J. Neurochem.* 136 172–185. 10.1111/jnc.13392 26465922

[B8] Brundin P. Dave K. D. Kordower J. H. (2017). Therapeutic approaches to target alpha-synuclein pathology. *Exp. Neurol.* 298(Pt B), 225–235. 10.1016/j.expneurol.2017.10.003 28987463 PMC6541231

[B9] El-Agnaf O. Overk C. Rockenstein E. Mante M. Florio J. Adame A. (2017). Differential effects of immunotherapy with antibodies targeting alpha-synuclein oligomers and fibrils in a transgenic model of synucleinopathy. *Neurobiol. Dis.* 104 85–96. 10.1016/j.nbd.2017.05.002 28476636 PMC5954414

[B10] Fellner L. Irschick R. Schanda K. Reindl M. Klimaschewski L. Poewe W. (2013). Toll-like receptor 4 is required for alpha-synuclein dependent activation of microglia and astroglia. *Glia* 61 349–360. 10.1002/glia.22437 23108585 PMC3568908

[B11] Fellner L. Jellinger K. A. Wenning G. K. Stefanova N. (2011). Glial dysfunction in the pathogenesis of alpha-synucleinopathies: emerging concepts. *Acta Neuropathol.* 121 675–693. 10.1007/s00401-011-0833-z 21562886 PMC4730553

[B12] Fellner L. Stefanova N. (2013). The role of glia in alpha-synucleinopathies. *Mol. Neurobiol.* 47 575–586. 10.1007/s12035-012-8340-3 22941028 PMC3589649

[B13] Fernagut P. O. Meissner W. G. Biran M. Fantin M. Bassil F. Franconi J. M. (2014). Age-related motor dysfunction and neuropathology in a transgenic mouse model of multiple system atrophy. *Synapse* 68 98–106. 10.1002/syn.21719 24243499

[B14] Games D. Valera E. Spencer B. Rockenstein E. Mante M. Adame A. (2014). Reducing C-terminal-truncated alpha-synuclein by immunotherapy attenuates neurodegeneration and propagation in Parkinson’s disease-like models. *J. Neurosci.* 34 9441–9454. 10.1523/JNEUROSCI.5314-13.2014 25009275 PMC4087215

[B15] Kahle P. J. Neumann M. Ozmen L. Muller V. Jacobsen H. Spooren W. (2002). Hyperphosphorylation and insolubility of alpha-synuclein in transgenic mouse oligodendrocytes. *EMBO Rep.* 3 583–588. 10.1093/embo-reports/kvf109 12034752 PMC1084143

[B16] Ko W. K. D. Bezard E. (2017). Experimental animal models of Parkinson’s disease: a transition from assessing symptomatology to alpha-synuclein targeted disease modification. *Exp. Neurol.* 298 172–179. 10.1016/j.expneurol.2017.07.020 28764902

[B17] Koga S. Dickson D. W. (2017). Recent advances in neuropathology, biomarkers and therapeutic approach of multiple system atrophy. *J. Neurol. Neurosurg. Psychiatry* 89 175–184. 10.1136/jnnp-2017-315813 28860330

[B18] Koprich J. B. Kalia L. V. Brotchie J. M. (2017). Animal models of alpha-synucleinopathy for Parkinson disease drug development. *Nat. Rev. Neurosci.* 18 515–529. 10.1038/nrn.2017.75 28747776

[B19] Kovacs G. G. Wagner U. Dumont B. Pikkarainen M. Osman A. A. Streichenberger N. (2012). An antibody with high reactivity for disease-associated alpha-synuclein reveals extensive brain pathology. *Acta Neuropathol.* 124 37–50. 10.1007/s00401-012-0964-x 22370907

[B20] Lee J. S. Lee S. J. (2016). Mechanism of anti-alpha-synuclein immunotherapy. *J. Mov. Disord.* 9 14–19. 10.14802/jmd.15059 26828212 PMC4734990

[B21] Lindstrom V. Fagerqvist T. Nordstrom E. Eriksson F. Lord A. Tucker S. (2014). Immunotherapy targeting alpha-synuclein protofibrils reduced pathology in (Thy-1)-h[A30P] alpha-synuclein mice. *Neurobiol. Dis.* 69 134–143. 10.1016/j.nbd.2014.05.009 24851801

[B22] Lopes da Fonseca T. Villar-Pique A. Outeiro T. F. (2015). The interplay between alpha-synuclein clearance and spreading. *Biomolecules* 5 435–471. 10.3390/biom5020435 25874605 PMC4496680

[B23] Mandler M. Valera E. Rockenstein E. Mante M. Weninger H. Patrick C. (2015). Active immunization against alpha-synuclein ameliorates the degenerative pathology and prevents demyelination in a model of multiple system atrophy. *Mol. Neurodegener.* 10:10. 10.1186/s13024-015-0008-9 25886309 PMC4411775

[B24] Mandler M. Valera E. Rockenstein E. Weninger H. Patrick C. Adame A. (2014). Next-generation active immunization approach for synucleinopathies: implications for Parkinson’s disease clinical trials. *Acta Neuropathol.* 127 861–879. 10.1007/s00401-014-1256-4 24525765 PMC4034750

[B25] Masliah E. Rockenstein E. Mante M. Crews L. Spencer B. Adame A. (2011). Passive immunization reduces behavioral and neuropathological deficits in an alpha-synuclein transgenic model of Lewy body disease. *PLoS One* 6:e19338. 10.1371/journal.pone.0019338 21559417 PMC3084838

[B26] Refolo V. Bez F. Polissidis A. Kuzdas-Wood D. Sturm E. Kamaratou M. (2018). Progressive striatonigral degeneration in a transgenic mouse model of multiple system atrophy: translational implications for interventional therapies. *Acta Neuropathol. Commun.* 6:2. 10.1186/s40478-017-0504-y 29298733 PMC5753576

[B27] Rockenstein E. Ostroff G. Dikengil F. Rus F. Mante M. Florio J. (2018). Combined active humoral and cellular immunization approaches for the treatment of synucleinopathies. *J. Neurosci.* 38 1000–1014. 10.1523/JNEUROSCI.1170-17.2017 29246926 PMC5783958

[B28] Sanchez-Guajardo V. Febbraro F. Kirik D. Romero-Ramos M. (2010). Microglia acquire distinct activation profiles depending on the degree of alpha-synuclein neuropathology in a rAAV based model of Parkinson’s disease. *PLoS One* 5:e8784. 10.1371/journal.pone.0008784 20098715 PMC2808388

[B29] Sanchez-Guajardo V. Tentillier N. Romero-Ramos M. (2015). The relation between alpha-synuclein and microglia in Parkinson’s disease: recent developments. *Neuroscience* 302 47–58. 10.1016/j.neuroscience.2015.02.008 25684748

[B30] Spencer B. Valera E. Rockenstein E. Overk C. Mante M. Adame A. (2017). Anti-alpha-synuclein immunotherapy reduces alpha-synuclein propagation in the axon and degeneration in a combined viral vector and transgenic model of synucleinopathy. *Acta Neuropathol. Commun.* 5:7. 10.1186/s40478-016-0410-8 28086964 PMC5237270

[B31] Stefanova N. (2017). Translational therapies for multiple system atrophy: bottlenecks and future directions. *Auton. Neurosci.* 211 7–14. 10.1016/j.autneu.2017.09.016 29017831

[B32] Stefanova N. Fellner L. Reindl M. Masliah E. Poewe W. Wenning G. K. (2011). Toll-like receptor 4 promotes alpha-synuclein clearance and survival of nigral dopaminergic neurons. *Am. J. Pathol.* 179 954–963. 10.1016/j.ajpath.2011.04.013 21801874 PMC3157205

[B33] Stefanova N. Georgievska B. Eriksson H. Poewe W. Wenning G. K. (2012). Myeloperoxidase inhibition ameliorates multiple system atrophy-like degeneration in a transgenic mouse model. *Neurotox. Res.* 21 393–404. 10.1007/s12640-011-9294-3 22161470

[B34] Stefanova N. Reindl M. Neumann M. Haass C. Poewe W. Kahle P. J. (2005). Oxidative stress in transgenic mice with oligodendroglial alpha-synuclein overexpression replicates the characteristic neuropathology of multiple system atrophy. *Am. J. Pathol.* 166 869–876. 10.1016/S0002-9440(10)62307-3 15743798 PMC1602361

[B35] Stefanova N. Reindl M. Neumann M. Kahle P. J. Poewe W. Wenning G. K. (2007). Microglial activation mediates neurodegeneration related to oligodendroglial alpha-synucleinopathy: implications for multiple system atrophy. *Mov. Disord.* 22 2196–2203. 10.1002/mds.21671 17853477

[B36] Stefanova N. Wenning G. K. (2015). Animal models of multiple system atrophy. *Clin. Auton. Res.* 25 9–17. 10.1007/s10286-014-0266-6 25585910 PMC4412689

[B37] Steiner J. A. Quansah E. Brundin P. (2018). The concept of alpha-synuclein as a prion-like protein: ten years after. *Cell Tissue Res.* 10.1007/s00441-018-2814-1 [Epub ahead of print]. 29480459 PMC6541204

[B38] Tenreiro S. Reimao-Pinto M. M. Antas P. Rino J. Wawrzycka D. Macedo D. (2014). Phosphorylation modulates clearance of alpha-synuclein inclusions in a yeast model of Parkinson’s disease. *PLoS Genet.* 10:e1004302. 10.1371/journal.pgen.1004302 24810576 PMC4014446

[B39] Venezia S. Refolo V. Polissidis A. Stefanis L. Wenning G. K. Stefanova N. (2017). Toll-like receptor 4 stimulation with monophosphoryl lipid A ameliorates motor deficits and nigral neurodegeneration triggered by extraneuronal alpha-synucleinopathy. *Mol. Neurodegener.* 12:52. 10.1186/s13024-017-0195-7 28676095 PMC5496237

[B40] Villadiego J. Labrador-Garrido A. Franco J. M. Leal-Lasarte M. De Genst E. J. Dobson C. M. (2018). Immunization with alpha-synuclein/Grp94 reshapes peripheral immunity and suppresses microgliosis in a chronic Parkinsonism model. *Glia* 66 191–205. 10.1002/glia.23237 29024008

[B41] Wilhelm I. Nyul-Toth A. Suciu M. Hermenean A. Krizbai I. A. (2016). Heterogeneity of the blood-brain barrier. *Tissue Barriers* 4:e1143544. 10.1080/21688370.2016.1143544 27141424 PMC4836475

[B42] Winkler E. A. Sengillo J. D. Bell R. D. Wang J. Zlokovic B. V. (2012). Blood-spinal cord barrier pericyte reductions contribute to increased capillary permeability. *J. Cereb. Blood Flow Metab.* 32 1841–1852. 10.1038/jcbfm.2012.113 22850407 PMC3463878

[B43] Wong Y. C. Krainc D. (2017). alpha-synuclein toxicity in neurodegeneration: mechanism and therapeutic strategies. *Nat. Med.* 23 1–13. 10.1038/nm.4269 28170377 PMC8480197

[B44] Zhang W. Wang T. Pei Z. Miller D. S. Wu X. Block M. L. (2005). Aggregated alpha-synuclein activates microglia: a process leading to disease progression in Parkinson’s disease. *FASEB J.* 19 533–542. 10.1096/fj.04-2751com 15791003

