# Supplementary figures and images for: Region-Specific Effects of Immunotherapy With Antibodies Targeting α-synuclein in a Transgenic Model of Synucleinopathy
Source: Front Neurosci. 2018 Jul 4;12:452. doi: 10.3389/fnins.2018.00452 (PMC6039792; doi:10.3389/fnins.2018.00452)

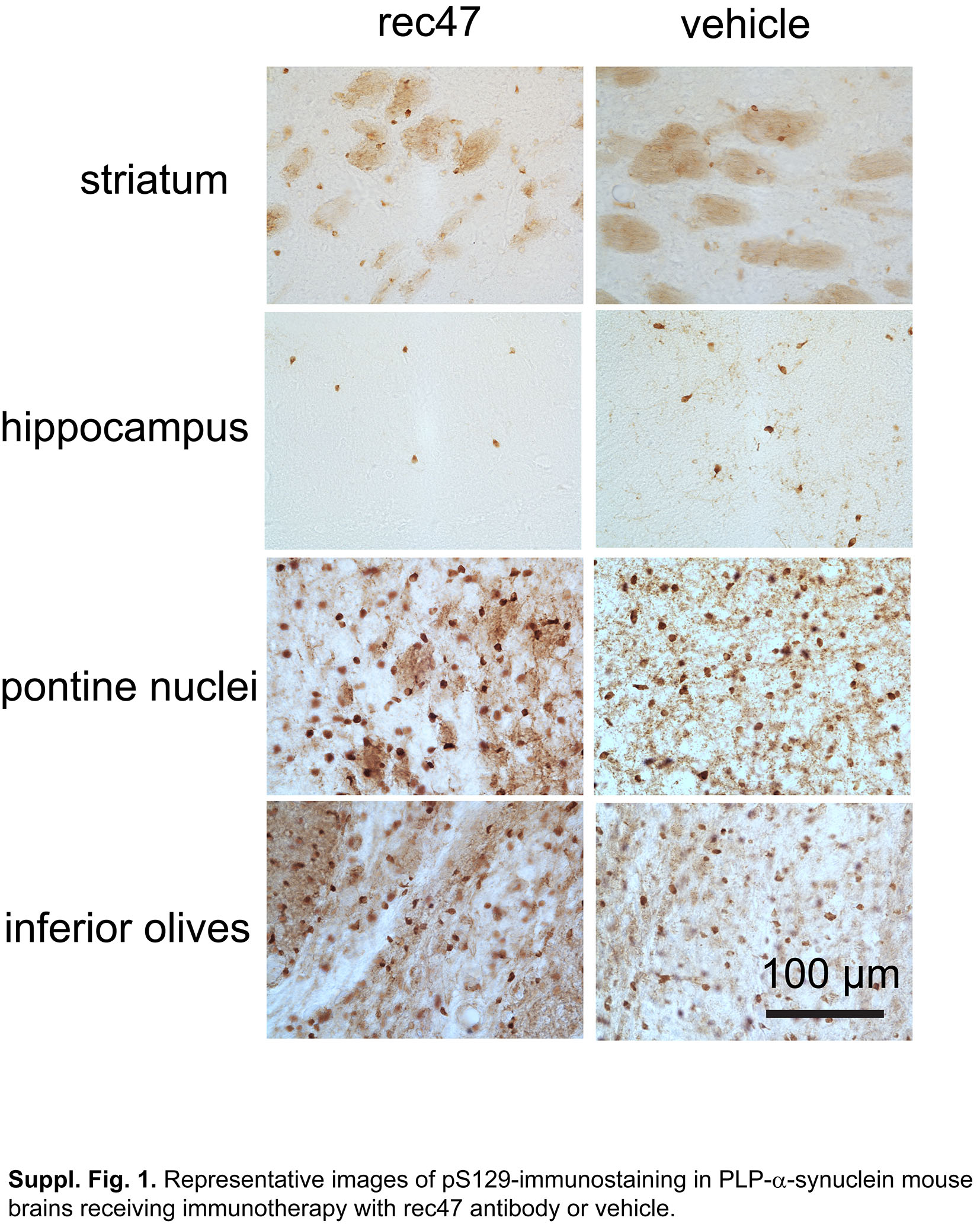

Supplement: Supplementary file 1 [file Image_1.JPEG]
